# Supplementary material for: Automated identification of piglet brain tissue from MRI images using Region-based Convolutional Neural Networks
Source: PLoS One. 2023 May 11;18(5):e0284951. doi: 10.1371/journal.pone.0284951 (PMC10174584; doi:10.1371/journal.pone.0284951)
Supplement: Attachment — Submitted filename: response_to_reviewer.docx [file pone.0284951.s001.docx]

We are very grateful for the helpful comments provided by the reviewer. We have addressed the points that have been raised and we feel that the manuscript has been improved.

**Reviewer #1: CNNs for automated brain extraction abound for human brains, but these methods have apparently not yet been developed for piglet brains. This is a reasonable application of deep learning technology. However, there are multiple major methodological criticisms that should be addressed, including:**

**1. Low number of subjects. 27 subjects is a low number to train on, although a model could possibly perform reasonably well. However, 5 test subjects is definitely not enough for a reasonable evaluation.**

We agree that more test subjects are required than were presented in the original manuscript. In the revised manuscript, we performed Nested Cross-Validation, which has been shown to provide non-biased accuracy estimates, even when a small number of samples are available. (Vabalas A, Gowen E, Poliakoff E, Casson AJ. Machine learning algorithm validation with a limited sample size. PLoS One. 2019;14(11):e0224365.**)** Using this method, we sorted all pigs into six test groups. Each group was tested using a model created from the remaining pigs. Therefore, each of the 32 pigs was used as a test case.

**2. While mask-RCNN has been used for segmentation, a 3D u-net is generally superior for brain extraction. The Unet is a state of the art biomedical image segmentation tool that may be helpful to the authors for getting higher performance. It might also be interesting to compare different architectures. Also there should be a reason why the authors use a 2D network rather than a multi slice (2.5D) or 3D network, which has been shown to perform better.**

We agree that the literature provides multiple examples of U-Net being successfully applied to skull-stripping or brain extraction. The literature comparing Mask R-CNN with U-Net is active, and we believe that a comparison of the two approaches using our data would be valuable, but we feel that it is beyond the scope of the current paper.

We have chosen a 2D architecture because it is computationally less demanding, and because the manual segmentations were performed using 2D. Because the 2D manual segmentations are used as the “gold standard” for cross-validation, deviations from this could be difficult to interpret, because they could arise from improvements in segmentation accuracy. We therefore decided to start with 2D as the ideal baseline comparison with manual segmentation, before adding more complexity for greater comparisons.

**3. Some more details are needed about the training. For example, what was the batch size and how many epochs were used for training?**

These details have been added to the paper.

**4. Performance measures should use more than just volume estimates and correlations. Specifically, at least Dice scores should be reported for evaluation of the segmentations.**

We agree with the reviewer that additional measures are needed. We have updated the manuscript to report Dice coefficients.

**5. There are also some very confusing parts of the paper. For example, "Model A" and "Model B" are only referred to in the Figure 3 legend (and in Figure 4). I'm not sure I see what these two models refer to from the Methods or Results section.**

We have revised the paper to clarify that the function of the different models. The new draft includes six models, labelled A through F, used for Nested Cross-Validation. We have carefully reviewed and revised the paper to improve clarity.

Finally, we note that the original paper included an example of brain mask where the machine-generated solution was more accurate than the manually traced mask. This was found to be an erroneous example based on a file identification error; the flawed manually-traced mask had already been corrected before any of the training took place. This example has been removed from the manuscript.
