# Supplementary material for: Automated identification of piglet brain tissue from MRI images using Region-based Convolutional Neural Networks
Source: PLoS One. 2023 May 11;18(5):e0284951. doi: 10.1371/journal.pone.0284951 (PMC10174584; doi:10.1371/journal.pone.0284951)
Supplement: Attachment — Submitted filename: reviewer_comments_responses.docx [file pone.0284951.s002.docx]

Reviewer #1: Overall, the segmentation results appear to be very good, and the cross-fold validation study is rigorous. Splitting the folds over pigs (subject-wise split) avoids data contamination issues between training and testing, which is good.

However, I do not like how the outlier case of one pig was manually-corrected and then the segmentation values were used to report final Dice scores. Manual-correction is fine, but I feel that this is a biased presentation of results. Instead, I encourage you to report both the Dice overlap scores using the original data (that includes the failure case) as well as the results after manual correction for full transparency of results. Imaging artefacts happen all the time, and I think presenting these full results would be more useful for readers.

**We thank the reviewer for their helpful review. We agree with the reviewer and we have revised the manuscript to report Dices scores before and after this manual correction.**

What form of image intensity normalization was performed? Intensity normalization for MR images is often a critical component. It might not be problem here since all images were from the same scanner, but it would be problem if different scanners were used (or the same scanner was used after any upgrades). Intensity normalization could also help to control for intensity outliers (as seen in your failure case). Please elaborate on any normalization methods used.

**We agree with the reviewer on the importance of intensity. We have revised the manuscript to state that we did not perform normalization, and to state that might have prevented the segmentation error mentioned by the reviewer, and that it would likely be important for use with data from other scanning conditions.**

Largest connected component filtering was performed as a post-processing step. It would be helpful to report the Dice segmentation values prior to post-processing as well. This would give readers a sense of how well the original segmentation performed compared to how much the post-processing helped.

**We have updated the manuscript to include this information, and to quantify the improvements in Dice coefficients due to largest connected component filtering**

The inclusion of Dice overlap as similarity metric is good and gives a measure of overall segmentation performance. But, typically, Dice by itself is not the only metric used for segmentation evaluation. Other metrics that are commonly used are Harsdorff Distance (HD) and Mean Surface Distance. In particular, HD is of interest because this gives a measure of worst-case segmentation performance. Worst case segmentation performance is often of interest to readers.

**We have updated the manuscript to report Hausdorff Distance measures.**

Training and inference used 2D image slices. During inference, all 2D slices of a piglet were combined into a 3D volume. This has the potential to result in mask results that are not smooth at the boundary because each 2D slice is independent of its neighbors (this is where 2.5D or 3D processing would help). Please discuss the spatial consistency of results between neighboring slices within the 3D volume.

**We have updated the manuscript with a discussion of the potential advantages of 3D processing for improvement of consistency between slices.**

Grammar/Typographical:
Line 134: “all by the” -> “all but the”

**Thank you for pointing this out; it has been corrected.**

Reviewer #2: I have the following concerns about this manuscript:
1- This manuscript is written by a way different from usual known articles including sectioning and subsectioning, references ..etc.

**We thank the reviewer for their review and comments. We believe that the sectioning is consistent with the usual pattern, including Introduction, Methods, Results, and Discussion. The Methods section is sub-sectioned into Animals and Care Practices, MRI Acquisition, Manual Brain Extraction, Automated Brain Extraction, and Validation.**

2- There is no mathematical or statistical analysis at all.

**We have updated the manuscript to include Hausdorff Distance values, and some descriptive statistics of them.**

3- The methodology used in training, validation, and testing the used model is unclear.

**We tested the model using nested cross-validation. We do not have a separate validation set because we did not perform hyperparameter tuning. The method of nested cross-validation and the potential usefulness of hyperparameter tuning is described in the paper.**

4- The author should compare their results with related published references performing the segmentation task using the same data set without restriction themselves to the piglet MRI images.

**We agree that the inclusion of more data could strengthen the manuscript; however, we believe that the inclusion of non-piglet MRI images is beyond the scope of the manuscript.**
