# Supplementary material for: Automated identification of piglet brain tissue from MRI images using Region-based Convolutional Neural Networks
Source: PLoS One. 2023 May 11;18(5):e0284951. doi: 10.1371/journal.pone.0284951 (PMC10174584; doi:10.1371/journal.pone.0284951)
Supplement: Attachment — Submitted filename: Response to Reviewers.docx [file pone.0284951.s003.docx]

Reviewer #1: The authors have addressed my comments.

The main limitation of the paper’s evaluation is that the segmentation approach is not compared to alternative segmentation methods as a benchmark. For example, comparison to the 3D U-net approach in [12] using the data from this paper would be ideal. The method in [12] is another segmentation approach applied to very similar data, piglet brain MRI. The proposed approach to use Mask R-CNN would be rigorously justified with a direct head-to-head comparison with the 3D U-net using your dataset.

Absent this numerical comparison, additional discussion comparing to the U-net approach to the proposed approach would be helpful in the Discussion section. The performance of [12] is briefly mentioned in the Discussion (line 223), but a more thorough comparison to the proposed method (both strengths and weaknesses) may be warranted since it is a such a similar application area. However, this discussion must note that a true comparison cannot be made because the two approaches used different datasets.

***Authors: We are pleased that the reviewer has found our responses to be suitable. We agree that the paper can benefit from additional comparisons with Ref. [12]. We have added several sentences comparing various aspects of the two methods, including network architectures, 2D vs. 3D, and post-processing. We comment on the relative strengths and weaknesses of these approaches. We have also referenced additional literature comparing the architectures used in the two studies (Mask R-CNN vs. U-Nets). We have also noted the differences in Dice Cofficients and Hausdorff Distances between the studies, and we have noted that an accurate comparison of the two methods would require the use of the same datasets. We conclude these comparisons with the statement that our results suggest that our 2D Mask R-CNN approach can be suitable for this application.***

Reviewer #2: It is the second time to review this manuscript, and almost all my concerns didn't addressed:

1- This manuscript is written by a way different from usual known articles including
sectioning and subsectioning, references ..etc.

Author Response: We thank the reviewer for their review and comments. We believe that the
sectioning is consistent with the usual pattern, including Introduction, Methods,
Results, and Discussion. The Methods section is sub-sectioned into Animals and Care
Practices, MRI Acquisition, Manual Brain Extraction, Automated Brain Extraction, and
Validation.

Reviewer again: Where is the "Conclusion" section?
Please, see one of the papers published y PloS One:

Zhai Y, Davenport B, Schuetz K, Pappu HR (2022) An on-site adaptable test for rapid and sensitive detection of Potato mop-top virus, a soilborne virus of potato (Solanum tuberosum). PLoS ONE 17(8): e0270918.
[https://doi.org/10.1371/journal.pone.0270918](https://urldefense.com/v3/__https:/doi.org/10.1371/journal.pone.0270918__;!!DZ3fjg!4Ewv3Zkr3OxnGL97xiYpwUPg_rupo2H7_miO_ea3Kugyy_OGU9kouINX4qjlvmKh6_4zqGng-R9g5HCK3w$)
---------------------------------

***Authors: We thank the reviewer for clarifying the comment. We agree that a Conclusion is typically helpful. However, in the PLOS One “Submission Guidelines” under “Manuscript Organization” it states that a “Conclusions” section is optional. We prefer to not have a separate conclusion section; however, in response to the reviewers concern, we moved two summary sentences from earlier in the discussion to the beginning of the final paragraph of the paper, to improve readability.***

***https://journals.plos.org/plosone/s/submission-guidelines#loc-style-and-format***

2- There is no mathematical or statistical analysis at all.
Response: We have updated the manuscript to include Hausdorff Distance values, and
some descriptive statistics of them.

Reviewer again: Not addressed
The authors can say that they didn't have any mathematical analysis since they are doing empirical research, then the editorial staff take their decision.

***Authors: It is not clear to us what additional mathematical or statistical analysis the reviewer may be suggesting.***

3- The methodology used in training, validation, and testing used in the model is unclear.

Response: We tested the model using nested cross-validation. We do not have a
separate validation set because we did not perform hyperparameter tuning. The
method of nested cross-validation and the potential usefulness of hyperparameter
tuning is described in the paper.

Reviewer again: point Addressed

***Authors: We are pleased that the reviewer has found our response to be suitable.***

4- The authors should compare their results with related published references
performing the segmentation task using the same data set without restriction
themselves to the piglet MRI images.

Reviewer again: Not addressed
My opinion is: The comparison with the state-of-the-art is necessary or at least the authors should describe why their approach gives sufficient results that will e an added value to this research area without evidences!. Especially the tool used in this research (Mask R-CNN) is not new!

***Authors: We have revised the manuscript to provide a more detailed discussion of how our method compares to a similar study in piglets that was recently published. The expanded text emphasizes that although the two studies used rather different approaches, (uNet vs Mask R-CNN, 3D vs 2D, more and less post-processing), the accuracy of the extractions appears to be comparable. We believe that demonstration of suitable accuracy using a method that has not previously been employed for the application is indeed a solid contribution to the field. Moreover, we will be sharing the data and the code at the time of publication to facilitate any future head-to-head comparisons that may be of interest.***

-------------------
New sample comments:
A- Line 17, the first sentence in the "Abstract" : Magnetic Resonance Imaging.... >>> Magnetic Resonance Imaging (MRI)....

***Authors: We believe it is conventional for acronyms to not be used and defined in the Abstract. We have defined this acronym on line 33 of the Introduction. Nevertheless, this comment from the reviewer helped us to recognize a capitalization inconsistency between the Abstract and the Introduction, and we have revised “Magnetic Resonance Imaging” in the Abstract to “Magnetic resonance imaging”.***

B- How can we evaluate the automated technique visually?
Line 158 : "Visual inspection of the brain extractions reveals good accuracy of automatic brain extractions
(Fig 3)....

***Authors: Visual inspection by a human is valuable tool for evaluation. These inspections revealed segmentations issues that are discussed in the paper. Indeed, the “gold standard” method of brain extraction is based on human inspection.***
